# Supplementary material for: Pain scores reduction with the use of ultrasound-guided paracervical nerve block in patients with cervical cancer undergoing intracavitary brachytherapy: A randomized controlled trial
Source: PLoS One. 2024 Oct 24;19(10):e0310238. doi: 10.1371/journal.pone.0310238 (PMC11500884; doi:10.1371/journal.pone.0310238)
Supplement: Attachment — Submitted filename: Response to Reviewers.docx [file pone.0310238.s003.docx]

2024/4/2

Marianne Clemence

Staff Editor

*PLoS One*

Dear Editors:

We wish to re-submit the manuscript entitled “Pain score reduction with the use of ultrasound-guided paracervical nerve block in patients with cervical cancer undergoing intracavitary brachytherapy: A randomized controlled trial.” The manuscript ID is PONE-D-23-40504.

We thank you and the reviewers for your thoughtful suggestions and insights. The manuscript has benefited from these insightful suggestions. I look forward to working with you and the reviewers to move this manuscript closer to publication in the *PLoS One*.

We have studied the comments carefully and have made the necessary corrections in accordance with the reviewers’ suggestions. Revised portions of text are marked in red in the manuscript. The responses to all comments have been prepared and given below.

Thank you for your consideration. I look forward to hearing from you.

Sincerely,

Chao Zhou,

Department of Anesthesiology, The Fourth Hospital of Hebei Medical University

zhouchao870607@163.com

+8618531179002

Response to Journal Requirements:

*Q1: Please ensure that your manuscript meets PLOS ONE's style requirements, including those for.*

Response: We have checked the manuscript formatting and file naming protocols to ensure compliance with *PLOS ONE's* style requirements. If any errors remain, please do not hesitate to inform us.

*Q2: We note that the protocol uploaded with your submission is written in the past tense and includes exact study dates. Could you please confirm that the clinical trial protocol you have included as Supplementary Information is the version that was submitted to and approved by your ethics committee before the trial began?*

Response: In the newly uploaded translated, we have corrected the tense issues. Additionally, we have provided the original version of the research protocol as evidence. We confirm that the clinical trial protocol included as Supplementary Information is the version that underwent submission to to and approval by our ethics committee before the commencement of the trial.

Responses to the reviewer's comments:

Reviewer ＃1:

*Q1:* *Page 2 Line 24. Not sure why the labels L and C are chosen for the Test and Control treatments – although the reason for C as control is obvious (see comments on Table 1 below).*

Response: Accordingly, we have changed the label of the test group to “T,” corresponding to “Test,” to make it easier for readers to understand.

*Q2: Page 11 Line 190. As typed the expression for the sample size for one arm of the trial is not correct. The 1641.4 should be replaced by 0.5. See for example, Dobson and Gebski (1982), The Statistician, 35, 51-53. However, the sample size calculated of 40 per group (80 in all) is appropriate.*

Response: Thank you for highlighting this issue. We have conducted a thorough searched for related literature and have not found any errors in this formula. An image of the formula is shown below for reference. The discrepancy between the two formulas, despite producing identical results, stems from the unit used to represent the angle. In the original formula, the angle is measured in degrees. In response to your suggestion, we have modified the formula to make it easier for readers to understand.

Changes: $n=0.5\times\left[ \frac{(u_{\alpha}+u_{\beta})}{{sin}^{-1} \sqrt{p_{1}}-{sin}^{-1} \sqrt{p_{2}}} \right]^{2}$

*Q3: Page 11 Line 190.Note if the equation is to be quoted, definitions of what uα and uβ represent are required.*

Response: Accordingly, we have been defined uα，uβ and added description of uα，uβ on page 11 line 189-190.

Changes: *u_α_* and *u_β_* correspond to the *μ* value of Type I and Type II errors *,* respectively*, u*_α_ = 1.96; *u_β_* = 0.842.

*Q4: Page 12 Line 213 Table 1. Column 1 rows may be better labelled PCN, Test, Control or PCN, Yes, No.
Table 1 As the L and C groups are allocated at random, statistical testing is not required for comparing differences of age, BMI and ASA as, by definition, such differences are random. So, best to omit the P-value row.*

Response: Accordingly, we have revised the label name of the test group to “Group T” and deleted the p-value row.

*Q5: Table 1. As this is describing the patients, rather than the SD better to quote the minimum and maximum values of each variable.*

Response: After considering this advice, we think that SD can better reflect the distribution of the variable than the minimum and maximum values of each variable, and we have consulted several literatures related to nerve block published by *PLoS One*, in which SD is also used to describe the variation. However, if you think this item must be modified, please let us know, and we will adopt your opinions to make changes.

References:

[1] Kang Q, Wu L, Liu Y, Zhang X. Ultrasound-guided medial branch of the superior laryngeal nerve block to reduce peri-operative opioids dosage and accelerate patient recovery. PLoS One. 2023; 18 (12): e0295127. doi: 10.1371/journal.pone.0295127

[2] Zhipeng L, Meiyi H, Meirong W, Qunmeng J, Zhenhua J, Yuezhen H, et al. Ultrasound-guided internal branch of superior laryngeal nerve block on postoperative sore throat: A randomized controlled trial. PLoS One. 2020; 15 (11): e0241834. doi: 10.1371/journal.pone.0241834

*Q6: Page 12 Table 2. As the focus of the trial is on the difference L − C, this table should give these differences for each of T1 to T4, that is, -1.98, -1.08, -3.38 and -2.77. Then quote the 95% CI for each of these differences plus the P-values already quoted.Usual to quote P-values to 2 significant figures, hence 0.47, 0.68, 0.20 and 0.29. This comment also applies to other places in the text.*

Response: Accordingly, we have added the difference T – C and 95% CI for each of the these differences for T1 to T4. Furthermore, all p-values have been presented to two significant figures.

*Q7:* *Page* *12-13 Table 3. Same comments as for Table 2.*

Response: Accordingly, we have added the difference T – C and 95% CI for each of the these differences for T1 to T4. Furthermore, all p-values have been presented to two significant figures.

*Q8:* *Page* *13 Tables 4 & 5. I think it would be more helpful to quote the minimum and maximum scores rather than Q1 and Q3.*

Response: After taking this advice into account, we believe that Q1 and Q3 can reflect the distribution of the variable better than the minimum and maximum values of each variable. While deliberating this change, we have consulted several literatures related to nerve block published by *PLoS One*, in which IQR is also used to describe the variation. If you think this item must be modified, please let us know, and we will make the relevant changes.

References :

[3] Ranganath YS, Ramanujam V, Onodera Y, Qunmeng J, Zhenhua J, Yuezhen H, et al. Impact of paravertebral blocks on analgesic and non-analgesic outcomes after video-assisted thoracoscopic surgery: A propensity matched cohort study. PLoS One. 2021; 16. doi: 10.1371/journal.pone.0252059

*Q9:* *Page 14-15 Tables 6 & 7. More helpful to quote the minimum and maximum scores rather than Q1 and Q3.*

Response: After considering this advice, we believe that utilizing the Q1 and Q3 can provide a more accurate reflection of the variable's distribution compared to relying solely on the minimum and maximum values. During our deliberation on this adjustment, we referred to several studies on nerve blocks published by PLoS One, where the IQR was commonly employed for this description. If you still believe that this aspect necessitates modification, please inform us, and we will gladly incorporate your suggestions.

References:

[3] Ranganath YS, Ramanujam V, Onodera Y, Qunmeng J, Zhenhua J, Yuezhen H, et al. Impact of paravertebral blocks on analgesic and non-analgesic outcomes after video-assisted thoracoscopic surgery: A propensity matched cohort study. PLoS One. 2021; 16. doi: 10.1371/journal.pone.0252059

*Q10* *Page* *15 Table 7, row 2. Time taken by patients with high Alderete scores refers to a subset of the 80 patients, in which case the numbers concerned in each group need to be quoted here. Also, the difference in groups needs to be quoted with the 95% CI. Since the distribution of time is very skew, comparisons of medians are required. I do not know if these can be implemented in SPSS Statistics 23.0 which is used by the authors for their analysis.*

Response: Alderte score is a scoring system used to evaluate the recovery of patients after anesthesia, and the full score is 10 points. Alderte score ≥ 9 represents complete recovery of patients. Therefore, the Alderte score of all patients will be ≥ 9, and there are no patients with Alderte score <9. We analyzed the time for achieving an Aldrete score of ≥9 of the patients and modified the description of the Aldrete score in Table 7 to facilitate reader understanding. We have added the difference L – C (95% CI) of Alderte score in Table 7.

*Q11: Although, there is considerable interest in the differences between randomised groups at T1, T2, T3 and T4 the following overall comparison may be useful to include.*

Response: Accordingly, in Table 7, we have provided a general comparison of the operating times, Alderte score≥9 min, operator satisfaction, patient satisfaction, and patient willingness to return.

Reviewer #2:

*Q1: This type of treatment procedure has a very short duration, and in the past, we have achieved good results by combining short-term sedatives with low-dose analgesics. However, in this study, the two groups did not routinely use analgesic drugs, so it is reasonable for the control group to have a higher VAS score in T4*.

Response: In terms of analgesic drug administration, both groups were administered 0.03 mg/kg of nalbuphine for intravenous analgesia, as stated in the article on Page 8 Line 124.

*Q2: Compared to administering low-dose analgesics, ultrasound-guided paracervical nerve block requires additional equipment, operator capabilities, and more potential complications. Is it worth doing this in clinical work? Whether nerve block is better than local anesthesia or the use of low-dose analgesics should be more discussed. If a simpler method can be used to achieve the same effect, nerve block is obviously a more complex and risky operation.*

Response: While the ultra-guided parallel nerve block does require additional equipment and places higher demands on the operator, this study revealed notable benefits for patients:

1. Test group patients experienced a significant decrease in VAS score at T3 and T4.

2. At T2, there was a significant reduction in the body movement scores of the test group patients.

3. Both patient and operator satisfaction were high in the test group.

4. The test group exhibited a greater willingness to return.

5. Moreover, the parallel nerve block, completed under ultrasound guidance, offers the advantage of constant visualization of the puncture needle throughout the entire process of implementing the nerve block, avoiding complications. Notably, there were no nerve block related complications.

We have added the relevant information to the Discussion to further highlight the benefits of the method compared with alternative procedures, page 20-21, lines 302-314.

Changes: Research indicates that conscious sedation anesthesia can be used in patients with gynecological tumors undergoing ISBT treatment. However, when the sedation effect disappears and the instrument is removed, patients often exhibit moderate pain, subsequently requiring high doses of analgesics and potential psychosocial treatment [20]. Studies have also investigated a combination of propofol and ketamine for anesthesia. Although it can yield effective anesthesia, the subsequent incidence of nausea and vomiting in patients is high, which significantly increases their discomfort [21]. Moreover, following applicator placement, it necessitates transfer between the operating, imaging, and radiation therapy rooms. Such repeated movement can further increase the patient's experience of pain stimulation. Therefore, adopting the anesthesia method of remifentanil sedation combined with ultrasound-guided paracervical nerve block presents as a more suitable choice.

*Q3:  Is it common to use BIS monitoring for only 5 minutes of operation? Is it necessary or a waste of resources?*

Response: The use of BIS monitoring aimed to maintain uniform anesthesia depth across the two groups, mitigating potential outcome disparities due to different anesthesia depths. In clinical settings, the decision to use BIS monitoring is based on factors such as the patient's age and physical condition.
