# Supplementary material for: Pain scores reduction with the use of ultrasound-guided paracervical nerve block in patients with cervical cancer undergoing intracavitary brachytherapy: A randomized controlled trial
Source: PLoS One. 2024 Oct 24;19(10):e0310238. doi: 10.1371/journal.pone.0310238 (PMC11500884; doi:10.1371/journal.pone.0310238)
Supplement: Attachment — Submitted filename: Response_to_Reviewers.docx [file pone.0310238.s004.docx]

August 10, 2024

Marianne Clemence

Staff Editor

*PLoS One*

Dear Editors:

We wish to re-submit the manuscript entitled “Pain score reduction with the use of ultrasound-guided paracervical nerve block in patients with cervical cancer undergoing intracavitary brachytherapy: A randomized controlled trial.” The manuscript ID is PONE-D-23-40504.

We thank you and the reviewers for your thoughtful suggestions and insights. The manuscript has benefited from these insightful suggestions. I look forward to working with you and the reviewers to move this manuscript closer to publication in the *PLoS One*.

We have studied the comments carefully and have made the necessary corrections in accordance with the reviewers’ suggestions. Revised portions of the text are marked in the manuscript. The point-by-point responses to all comments have been prepared and given below.

Thank you for your consideration. I look forward to hearing from you.

Sincerely,

Chao Zhou,

Department of Anesthesiology, The Fourth Hospital of Hebei Medical University

zhouchao870607@163.com

+8618531179002

Response:

*Q1: Authors should expand the discussion part with additional content.*

Response: Thank you for your recommendation. We have supplemented the Discussion section and reorganized its content to make it easily understandable by the readers.

*Q2: In the Figure 2, swap the content in Chinese language with English or Mention a note below the figure. All the figure legends are very simple and authors should expand the legends with significance of the figure to the study.*

Response: Thank you for your advice. The Chinese content in the image is automatically generated by the ultrasound machine and has no meaning. However, to avoid any misunderstandings among readers, we have removed that from the image. In addition, we have expanded the figure legends for clarity.

*Q3: Mention trial number in the abstract: ChiCTR2300071580 [https://clin.larvol.com/trial-detail/ChiCTR2300071580].*

Response: Thank you for your suggestion. We have mentioned the clinical trial identifier in the Abstract.

*Q4: Conclusion should be explained vividly.*

Response: Thank you for your advice. We have partly rewritten the Discussion and provided sufficient explanations for the conclusions therein.

*Q5: Mention about the authors future directions depending on the results of this trial: For instance, Future trials could explore several avenues to build on these findings. Firstly, a larger sample size and multi-center trials would provide more robust data and generalizability. Additionally, investigating the long-term effects of paracervical nerve blocks on patient outcomes and quality of life would be valuable. Comparing the efficacy of ultrasound-guided paracervical nerve blocks with other pain management techniques could further refine treatment protocols. Moreover, assessing the cost-effectiveness and potential side effects in a more diverse patient population would be beneficial. Finally, integrating advanced imaging technologies and refining nerve block techniques could enhance precision and effectiveness in pain management for cervical cancer patients*

Response: Thank you for your recommendations. We have added our outlook on the research at the end of the Discussion section.
